# Supplementary material for: Causal Relationship between Aging and Anorexia Nervosa: A White-Matter-Microstructure-Mediated Mendelian Randomization Analysis
Source: Biomedicines. 2024 Aug 16;12(8):1874. doi: 10.3390/biomedicines12081874 (PMC11351342; doi:10.3390/biomedicines12081874)
Supplement: Supplementary file 1 [file biomedicines-12-01874-s001.zip › Supplementary Figure/Figure S8/Leaveoneout plot of MR analysis for TL to ubm-b-1594.pdf]

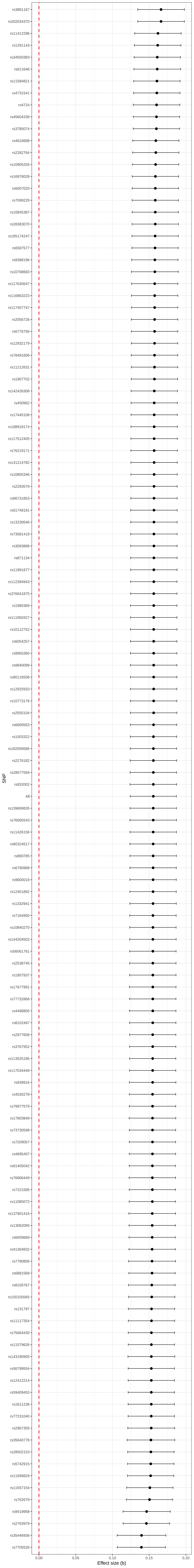

rs3891167
rs202034370
rs11412296
rs1291143
rs34550383
rs611646
rs11584821
rs4731541
rs4724
rs45604339
rs3785074
rs4616688
rs2282764
rs10905255
rs16978028
rs6007020
rs7099229
rs10845387
rs28363070
rs185174247
rs6587577
rs9398196
rs10768683
rs117630647
rs116863223
rs117407747
rs2056726
rs6776756
rs12932179
rs78491606
rs11212631
rs1907702
rs142426306
rs450962
rs17445108
rs188918174
rs117512405
rs76219171
rs141214782
rs10805346
rs2293579
rs66731853
rs61748181
rs13230646
rs73581419
rs3093888
rs871134
rs11991877
rs112394943
rs376641875
rs1985369
rs111950327
rs10112752
rs6054257
rs9955360
rs9940099
rs80116508
rs12925933
rs10773176
rs2555104
rs6669563
rs1003322
rs182059586
rs2276182
rs28577594
rs932002
All
rs139669835
rs76065543
rs11426156
rs80324517
rs869785
rs6790988
rs9600019
rs12451892
rs1332941
rs7164950
rs10840270
rs144204502
rs56061761
rs2538745
rs1957937
rs17677991
rs77732866
rs4498805
rs8102497
rs2977608
rs3767952
rs113525195
rs117034449
rs939916
rs4530278
rs79977579
rs17803849
rs73730598
rs7209057
rs4695407
rs61405042
rs76666449
rs7221585
rs11085072
rs137901416
rs13062095
rs6659669
rs41304832
rs7790856
rs6881568
rs8105767
rs150150565
rs131797
rs11117354
rs75664430
rs11579626
rs143190905
rs56799554
rs12412214
rs59409453
rs1611236
rs77231040
rs2967355
rs35640778
rs28502153
rs5742915
rs11699829
rs111557154
rs762679
rs9419958
rs2763979
rs35446936
rs77055526
SNP
0.00
0.05
0.10
0.15
0.20
Effect size (b)
